# Benzodioxane-benzamides targeting bacterial cell division protein FtsZ potentially disrupt SlmA-mediated nucleoid occlusion and reversible biomolecular condensation

**DOI:** 10.1016/j.ijbiomac.2025.148516

**Published:** 2025-10-23

**Authors:** Marta Sobrinos-Sanguino, Inés Barros-Medina, Lorenzo Suigo, Alessia Lanzini, Ermanno Valoti, William Margolin, Valentina Straniero, Begoña Monterroso, Silvia Zorrilla

**Affiliations:** a Department of Molecular and Cellular Biosciences, Centro de Investigaciones Biológicas Margarita Salas, Consejo Superior de Investigaciones Científicas (CSIC), 28040, Madrid, Spain; b Dipartimento di Scienze Farmaceutiche, Università degli Studi di Milano, Via Luigi Mangiagalli, 25, 20133, Milano, Italy; c Department of Microbiology and Molecular Genetics, McGovern Medical School, University of Texas, Houston, 77030, TX, USA; d Department of Crystallography and Structural Biology, Instituto de Química Física Blas Cabrera, Consejo Superior de Investigaciones Científicas (CSIC), 28006, Madrid, Spain

## Abstract

New strategies are urgently needed against antimicrobial resistance, a major health threat, and the different mechanisms regulating the bacterial cell division machinery offer multiple opportunities for developing novel therapeutics. FtsZ, an essential protein of this process, is targeted by multiple small molecules, and benzodioxane-benzamides (BDOBs) are among the most potent inhibitors in several bacterial species. BDOBs mechanisms are however poorly understood, particularly their impact on FtsZ’s interplay with partners and ability to assemble phase-separated biomolecular condensates potentially involved in stress sensing. We show that certain BDOBs shielded FtsZ against depolymerization induced by the nucleoprotein complexes of SlmA, which inhibit Z-ring formation near the nucleoid. In crowding cytomimetic conditions, BDOBs disrupted the canonical interconversion between FtsZ-SlmA condensates and polymers in response to GTP levels. Our results strongly suggest that BDOBs interfere with normal regulation of Z-ring assembly by antagonists such as SlmA that protect important cellular structures like the nucleoid. Specifically, BDOBs may reduce the susceptibility of FtsZ polymers to SlmA and impair biomolecular condensates reassembly. We propose that fine tuning of the equilibrium between FtsZ polymers and biomolecular condensates is important for spatial regulation of Z-rings and stress resistance, and this equilibrium is subverted by BDOBs.

## 1. Introduction

Antimicrobial resistance, currently among the most complicated global health challenges, threatens the ability of the available therapeutic arsenal to fight infections [1]. Because of its gradual increase in so many pathogens, together with other important factors such as re-emerging long-forgotten infections, infectious pathologies are expected to be the primary cause of disease-related death in the near future. The World Health Organization has placed antimicrobial resistance among the first ten worldwide health problems [2], and identified several Gram-negative pathogens as critical priority for the research and development of new antibiotics, among others *Acinetobacter baumannii* and Enterobacteriaceae. The latter group includes *Escherichia coli* (3GCR, third-generation cephalosporin-resistant; CR, carbapenem-resistant), based on their widespread prevalence and level and trend of resistance [3]. Overcoming this problem involves a multi-pronged approach like that conceptualized as One Health, which integrates human, animal, plant and environmental health as key areas for initiatives addressing the associated public health, economic and social implications [4]. Those efforts include increasing therapeutic efficiencies by development of new antimicrobial agents and the identification of new targets.

Proliferation and viability of bacteria critically depend on the optimal operation of their cell division machinery and the molecular mechanisms that spatiotemporally regulate the assembly of this machinery in coordination with other cell cycle events [5–8]. So far, research efforts for the development of novel strategies to curb bacterial infections by targeting cell division have mainly focused on the tubulin-like GTPase FtsZ [9–13]. This protein, conserved in most bacteria [14], is the central component of the Z-ring that, upon direct or indirect interaction with other division machinery elements, forms the divisome that coordinates membrane constriction and cell wall remodeling [5,15]. Many drugs from different chemical families have been developed that either block the GTP-dependent assembly of FtsZ into polymers or hyperstabilize them [13,16], ultimately interfering with functional divisome assembly and compromising cell viability. One prodrug is currently under clinical development (TXA709; [17–19]). The multiplicity of interactions of the division proteins and their regulators, among themselves and with the membrane and/or the bacterial chromosome, and their recently discovered assembly into dynamic biomolecular condensates by phase separation, represent promising and diverse new targets [20] that are still almost completely unexplored.

Formation of the Z-ring at the cell midpoint and future division site is achieved through the coordinated action of spatial regulatory mechanisms, one of them being nucleoid occlusion [6]. This mechanism blocks Z-ring assembly near the nucleoid with the purpose of avoiding DNA damage to any un-partitioned nucleoids by the invaginating septal wall. The main protein that mediates nucleoid occlusion in *E. coli* and other Gram-negatives, including most γ- and β-proteobacteria, is SlmA [21]. This protein directly interacts with FtsZ and with SlmA binding DNA sequences (SBS). These SBS are distributed throughout the chromosome except for the replication terminus (Ter) region, which is positioned near the cell midpoint during division. SlmA-SBS complexes strongly promote disassembly of GTP-triggered FtsZ polymers [22–24], and in cell-like crowded environments the ternary FtsZ-SlmA-SBS complexes form biomolecular condensates [25]. Notably, GTP triggers assembly of FtsZ polymers from these condensates that subsequently disassemble upon FtsZ polymer-driven GTP hydrolysis, leading to condensate reassembly [25].

Reversible assembly by phase separation of biomolecular condensates such as those formed by FtsZ-SlmA-SBS is emerging as a key principle for the organization and distribution of molecules within the bacterial cytoplasm, nucleoid and membrane [26–29]. It also plays crucial roles in the regulation of essential cell cycle processes, and has been related to stress responses and emergence of persister cell sub-populations tolerant to antibiotics [30–33]. As optimal functionality of biomolecular condensates largely relies on tight spatiotemporal regulation of their assembly/disassembly, alteration of these mechanisms may constitute a novel strategy to fight antimicrobial resistance [20,34]. Along this line, certain antimicrobial peptides have recently been shown to exert their killing activities through modulation of phase separation of nucleic acids [35,36].

Many of the compounds with demonstrated ability to perturb FtsZ assembly *in vitro* and *in vivo* belong to the benzamide family [12,13]. They bind to the interdomain cleft (IDC) of FtsZ, a cavity located within the globular domain and involving the T7 loop that activates FtsZ GTPase activity through its insertion into the adjacent monomer [9,12,37]. The absence of an analogous IDC region in eukaryotic tubulin represents an advantage for the development of low cytotoxicity compounds [12,13].

**Fig. 1.**
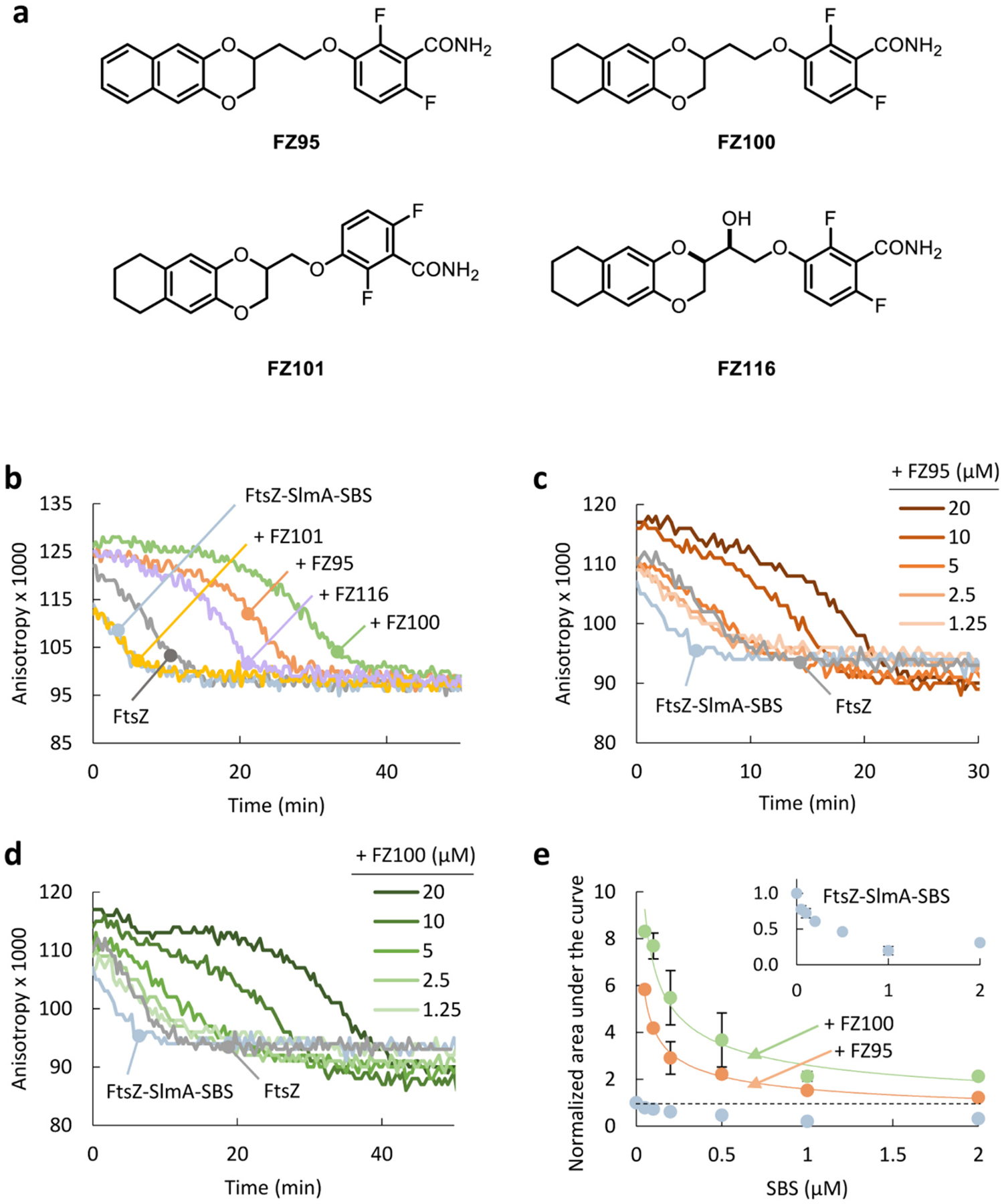
BDOBs FZ95, FZ100 and FZ116 hinder SlmA-SBS induced disassembly of GTP-triggered FtsZ polymers. a) Benzodioxane-benzamides used for the current study. b) Fluorescence anisotropy-based depolymerization profiles of FtsZ in the absence and presence of SlmA-SBS, with or without the specified compounds (20 μM). c, d) Effect of FZ95 and FZ100 concentration on the depolymerization of FtsZ-SlmA-SBS, monitored by anisotropy. Profiles without the compounds and those of FtsZ only, are shown for reference. e) Normalized area under the anisotropy curves of FtsZ depolymerization with different concentrations of SlmA-SBS (5:1 molar ratio) without and with 20 μM FZ95 or FZ100 (profiles in Fig. S1a, b). Inset shows a magnification of the values without compound. Some errors are within the data symbols. Areas were normalized to the values corresponding to FtsZ alone, indicated by a horizontal line. Data were recorded just after 1 mM GTP addition. Concentrations were 10 μM FtsZ (50 nM FtsZ-Alexa 488), 2 μM SlmA, 0.4 μM SBS, except when otherwise stated. Curves in (b-d) are representative of at least 3 replicates. Values in (e) are the average of 3 independent experiments ± SD. All experiments were performed in *dilute solution buffer* (see Materials and Methods).

Among the benzamides, 1,4-benzodioxane-benzamides (BDOBs) have emerged recently as a promising class of FtsZ inhibitors, displaying strong activity against Gram-positive species such as *Staphylococcus aureus* [38–42]. BDOBs also alter *E. coli* FtsZ assembly properties *in vitro* and strongly inhibit cell division of *E. coli* cells with defective efflux pumps or membrane permeability [43], which is of utmost interest given the relative scarcity of antimicrobials available for Gram-negatives [44]. Multiple BDOBs were tested in the recent study by Suigo et al. [43], many of which significantly reduced FtsZ GTPase activity and stabilized the filaments against depolymerization either in dilute solution or in crowding cytomimetic conditions. The most active BDOBs *in vitro* were FZ95, FZ100 and its *erythro* hydroxy derivative FZ116 (Fig. 1a), with an ethylenoxy linker between the two main moieties (the benzamide and the benzodioxane ring) and containing a 1,4-naphthodioxane ring (FZ95) or a 5,6,7,8-tetrahydro-1,4-naphthodioxane ring (FZ100 and FZ116). Compound FZ101, an inferior homologue of FZ100 showing a methylenoxy linker (Fig. 1a), only displayed modest activity in crowding conditions *in vitro*, where FtsZ polymers laterally interact forming bundles. When tested in efflux pump defective mutants, FZ95 and FZ116 were again among the most active and FZ101 was moderately active, whereas FZ100 killing activity was dependent on the specific efflux pump-deficient mutant evaluated. Despite these initial analyses, the precise mechanisms underlying the cell response to BDOBs remain largely unknown, in particular the possible implications of regulatory proteins interacting with FtsZ and of crowding-driven phase separation phenomena occurring in the cytoplasm.

In this work we explored the effect of selected BDOBs (FZ95, FZ100, FZ101 and FZ116, Fig. 1a) on the response of *E. coli* FtsZ polymers to the antagonist SlmA-SBS complexes and on the features of the crowding-driven FtsZ-SlmA-SBS biomolecular condensation. Using fluorescence anisotropy, fluorescence correlation spectroscopy (FCS), dynamic light scattering (DLS), confocal imaging and turbidity, we found that some BDOBs confer protection against the negative modulation of FtsZ polymerization by SlmA. Likewise, these compounds perturb the dynamic equilibrium between FtsZ-SlmA-SBS biomolecular condensates and polymers regulated by GTP. Our findings suggest that, as part of their antimicrobial mechanisms, BDOBs may interfere with the operation of systems ensuring nucleoid integrity under normal growth conditions. They may also reduce the ability of the cells to enter into dormancy by affecting the reversible sequestration of cell division proteins in biomolecular condensates.

## 2. Results

### 2.1. Benzodioxane-benzamides can confer resistance against FtsZ depolymerization induced by SlmA-SBS

Consistent with the role of SlmA as an antagonist of Z-ring formation, SlmA-SBS complexes promote disassembly of the FtsZ polymers induced by GTP [22,23]. To determine the impact of BDOBs on this inhibitory activity, we conducted fluorescence anisotropy experiments. GTP-triggered assembly of FtsZ polymers with FtsZ labeled with Alexa Fluor 488 (FtsZ-Alexa 488) as tracer increases anisotropy [45], a parameter sensitive to changes in size and/or flexibility of the fluorescent species, while depolymerization resulting from GTP hydrolysis and GDP accumulation lowers the observed anisotropy values ([43], and Fig. 1b). In the absence of the compounds, SlmA-SBS (2:0.4 μM) reduced FtsZ polymer lifetimes from ~15 min to ~10 min (Fig. 1b). The presence of FZ95, FZ100 or FZ116 (20 μM) in FtsZ-SlmA-SBS solutions delayed disassembly to >20 min, and FtsZ polymers lasted longer than without SlmA-SBS (Fig. 1b). In contrast, compound FZ101 did not have a significant effect on the negative regulation of FtsZ polymers by SlmA (Fig. 1b). Therefore, BDOBs FZ95, FZ100 and FZ116 counteract FtsZ polymer disassembly by SlmA-SBS, an effect not found for FZ101.

We next explored the impact of FZ95 and FZ100 at different concentrations of compounds and SlmA-SBS. Stabilization of FtsZ polymers against depolymerization induced by SlmA-SBS (2:0.4 μM) was enhanced with increasing concentrations of compound (Fig. 1c, d). The magnitude of the effect decreased at and below 5 μM compound, although significant suppression of SlmA effects was observed even at 1–2 μM compound. These concentrations are close to the MICs determined in AcrAB efflux pump-defective *E. coli* cells for FZ95 and FZ116, a derivative of FZ100 active *in vivo*. Interestingly, at 20 μM, both compounds counteracted the strong destabilization induced by SlmA-SBS at all concentrations of the complex tested: FtsZ polymer lifetime was longer than for FtsZ polymers on their own without compounds, and the overall effect was somewhat larger for FZ100 (Figs. 1e and S1a–d). Notably, the compounds did not completely block SlmA-SBS inhibition, as even the lowest concentration of SlmA-SBS resulted in a modestly shorter polymer duration compared with when only FtsZ polymers and the compounds were present (Fig. S1a, b). These experiments show that FZ95 and FZ100 are able to protect FtsZ polymers against SlmA-SBS induced depolymerization in a concentration dependent manner. Hyperstabilization of FtsZ polymers conferred by these compounds was sustained under all conditions tested, although the polymers were not completely insensitive to the antagonistic action of SlmA-SBS.

**Fig. 2.**
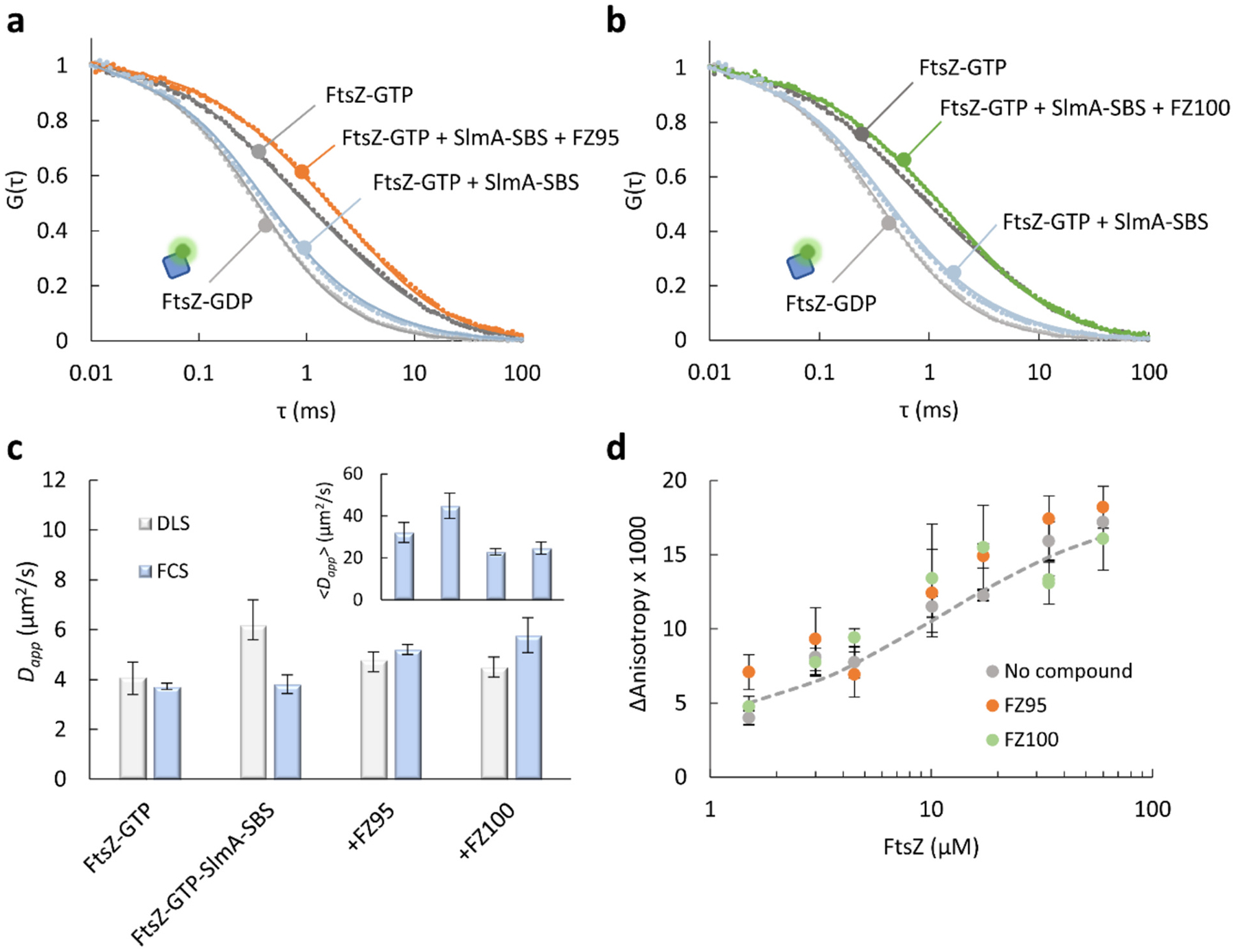
BDOBs FZ95 and FZ100 counteract the antagonistic effect of SlmA-SBS on GTP-triggered FtsZ polymers. a, b) Normalized FCS autocorrelation curves of FtsZ polymers (10 nM FtsZ-Alexa 488 as tracer) in the absence and presence of SlmA-SBS, without and with FZ95 (a) or FZ100 (b). Curves of FtsZ in the absence of GTP (oligomeric species, FtsZ-GDP) are shown for reference. Solid lines correspond to the fit of the models indicated in Materials and Methods. c) Apparent translational diffusion coefficients (*D*_*app*_) of FtsZ polymers measured by DLS and FCS corresponding to the slowest species (not entirely for FtsZ-GTP/SlmA-SBS by DLS) and, in the inset, average diffusion coefficients calculated from FCS data as specified in Materials and Methods. d) Fluorescence anisotropy-based binding titrations of SlmA (1 μM) and SBS-Fl (10 nM) with increasing concentrations of FtsZ (with 2 mM GTP), with or without 20 μM compounds. Dashed line corresponds to the fit of the model specified in Materials and Methods to the data without compound. Concentrations in (a-c) were 10 μM FtsZ, 2 μM SlmA, 0.4 μM SBS, 20 μM compounds, and 2 mM GTP, with an enzymatic regeneration system (RS). All experiments were performed in *dilute solution buffer* (see Materials and Methods). FCS data in (c) and anisotropy in (d) are the average of at least 3 independent replicates ± SD. DLS data are those obtained by simultaneous fit to 2–4 independent replicates ± SD.

To gain further insight into the effects of the compounds on the negative regulation of FtsZ polymerization by SlmA-SBS, we conducted FCS measurements using FtsZ-Alexa 488 as tracer and DLS measurements with the unlabeled protein. FCS autocorrelation profiles of FtsZ oligomeric species (apparent diffusion coefficient, *D*_*app*_, 53 ± 1 μm^2^/s) were shifted towards longer times by GTP (Fig. 2a, b), reflecting the formation of polymers (Fig. 2c). In the presence of SlmA-SBS (2:0.4 μM), FtsZ-GTP FCS autocorrelation curves approached those of non-polymeric FtsZ (Fig. 2a, b), because of the antagonistic action of the nucleoprotein complex on FtsZ polymerization, in agreement with previous data [22]. An important reduction in the fractional contribution of the slow-diffusing polymers to the autocorrelation function was observed, reflected in an increase in the <*D*_*app*_> (see Materials and Methods; Fig. 2c, inset), whereas the apparent diffusion coefficient of these large species was similar to that found in the absence of SlmA-SBS (Fig. 2c). With either FZ95 or FZ100, FCS profiles of FtsZ-GTP/SlmA-SBS were shifted to longer times (Fig. 2a, b), indicating higher prevalence of large species than for FtsZ-GTP/SlmA-SBS without the compounds, and even than for FtsZ-GTP alone, reflected in the decrease of the <*D*_*app*_> value (Fig. 2c, inset). *D*_*app*_ coefficients for the slow-diffusing polymers were slightly higher in the presence of the compounds than in their absence (Fig. 2c). DLS analysis showed that the presence of SlmA-SBS rendered somewhat larger *D*_*app*_ values, while addition of FZ95 or FZ100 induced a reduction in the diffusion to values closer to those determined for FtsZ-GTP in the absence of the inhibitor (Fig. 2c). FCS *D*_*app*_ values retrieved for the slow-diffusing species were similar to those determined by DLS for FtsZ-GTP (in agreement with previous reports [46]) and for FtsZ-GTP/SlmA-SBS when FZ95 or FZ100 were present, but significantly lower for FtsZ-GTP/SlmA-SBS in their absence (Fig. 2c). These higher DLS *D*_*app*_ values could reflect either a variation in the diffusion of the polymeric species (*i.e*., in their size and/or shape), or a large variation in the fraction of these species. The latter is more consistent with FCS analysis: although the main contribution to the DLS autocorrelation function comes typically from the larger species, the prevailing non-polymeric species in the presence of SlmA-SBS, according to FCS analysis (Fig. 2c, inset), could significantly influence the DLS autocorrelation curves. Collectively, these experiments further support that FZ95 and FZ100 protect FtsZ polymers from the disassembly induced by SlmA-SBS.

The inability of SlmA-SBS complexes to efficiently antagonize FtsZ polymerization in the presence of certain BDOBs could result from modification of the interaction of FtsZ polymers with the nucleoprotein complexes. To explore this possibility, we conducted fluorescence anisotropy binding titrations of SlmA-SBS, containing fluorescein-labeled SBS (SBS-Fl) as tracer, with increasing concentration of FtsZ, in the presence of GTP. The binding isotherm obtained without the compounds (Fig. 2d, apparent *ΔG* = 6.8 ± 0.1 kcal/mol) was consistent with those previously reported [22], and the presence of FZ95 or FZ100 did not substantially modify this behavior. FCS analysis using Alexa Fluor 488 labeled SBS (SBS-Alexa 488) showed a small shift of the profiles obtained for the oligonucleotide (*D* = 93 ± 9 μm^2^/s) upon binding to SlmA (*D* = 64.0 ± 0.1 μm^2^/s) and a further, more notable shift to longer times in the presence of FtsZ and GTP, indicative of binding of SlmA-SBS to the polymers (Fig. S1e). The FtsZ-GTP/SlmA-SBS profiles were very similar in the presence of FZ95 and FZ100. These observations suggest that shielding of FtsZ by these BDOBs may not arise from dramatic modification of SlmA-SBS binding to the FtsZ polymers, at least under the assayed conditions.

### 2.2. BDOBs can mildly modify FtsZ-SlmA-SBS condensates behavior

**Fig. 3.**
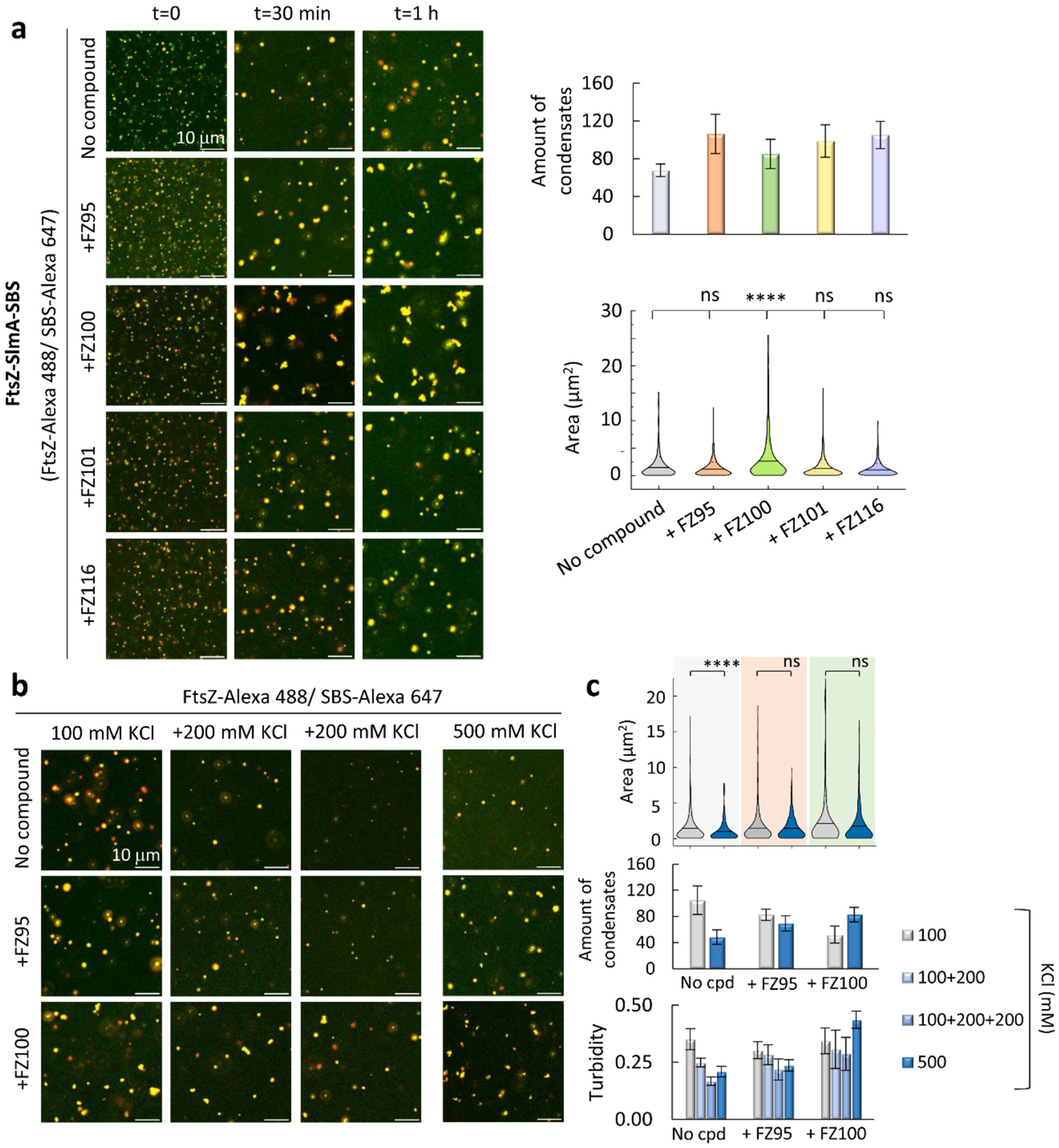
BDOBs can modestly affect FtsZ-SlmA-SBS biomolecular condensates. a) Merged confocal images showing time evolution of condensates without and with the indicated compounds (*t* = 0, freshly prepared condensates) and, on the right, average number per field and area of 30-min incubated condensates. b) Confocal images showing the effect of KCl addition in two consecutive steps to 30 min-incubated FtsZ-SlmA-SBS condensates in 100 mM KCl, without and with FZ95 and FZ100. On the far right, condensates prepared at 500 mM KCl. c) Area and average number per field of condensates at 100 or 500 mM KCl from experiments in (b). Below, turbidity of FtsZ-SlmA-SBS condensates (average from *n* ≥ 4 ± SD) at the specified KCl concentrations. Experiments were performed in *crowding conditions* (see Materials and Methods) with 150 g/L dextran (a) or in the same conditions with the specified KCl concentration (b, c). Concentrations were 10 μM FtsZ, 5 μM SlmA, 1 μM SBS and labeled elements, and 20 μM compounds. For statistical analysis (a, c) 6 images (82 × 82 μm fields) from at least two different experiments were analyzed. In (a), *n* = 406, 638, 511, 484 and 631 condensates for no compound, FZ95, FZ100, FZ101 and FZ116, respectively. In (c), *n* = 629, 495 and 314 for no compound, FZ95 and FZ100, at 100 mM KCl, respectively. *n* = 290, 417 and 497 for no compound, FZ95 and FZ100, at 500 mM KCl, respectively. ****p ≤ 0.0001, ns, not significant, by ANOVA on ranks test (Kruskal-Wallis) followed by Dunn’s test (a) or by Mann-Whitney Test (c). Solid line: mean, dashed lines: SD. Error bars correspond to SD.

To obtain further insight into the impact of BDOBs on the FtsZ-SlmA-SBS system, we investigated the response of the biomolecular condensates assembled by the two proteins and the DNA sites in crowding cytomimetic conditions [25] to these compounds. Confocal microscopy imaging showed that FtsZ-SlmA-SBS biomolecular condensates in the crowder dextran, with FtsZ-Alexa 488 and Alexa Fluor 647 labeled SBS (SBS-Alexa 647) as tracers, maintained their round shape during the time they were monitored (Fig. 3a). BDOBs (20 μM) had no detectable effect on condensate shape immediately after formation (Fig. 3a, *t* = 0). However, time evolution showed that while FZ100 did not significantly change the number of condensates, it appreciably deformed their shape after 30 min (Fig. 3a). The shape deformation was reduced using lower concentrations of FZ100 at this time point (Fig. S2a). With FZ95, FtsZ-SlmA-SBS round condensates were observed at 30 min, similar in size and slightly more abundant than in the absence of compounds (Figs. 3a and S2b). They coexisted with a minor fraction of somewhat deformed structures when incubated for 1 h (Fig. 3a). Using Ficoll instead of dextran as crowder, a similar deformation of condensates was found after 30 min incubation with FZ100, but not with FZ95 (Fig. S2c). FZ101 and FZ116 did not significantly modify condensate sizes in the time-interval studied, minimally increasing their number upon 30-min incubation (Fig. 3a). Colocalization of FtsZ and SBS was never lost, neither in the round structures nor in the irregular ones eventually formed with some of the compounds (Fig. 3a). These results suggest a time-dependent modification of FtsZ-SlmA-SBS condensates shape by FZ95, more evident for FZ100, while FZ101 and FZ116 did not have significant effects in the tests performed.

We next evaluated whether FZ95 and FZ100 could modify the response of condensates to ionic strength, which has a notable impact on FtsZ-SlmA-SBS condensation, probably related to the contribution of electrostatic interactions to their assembly [25]. In the absence of BDOBs, condensates were appreciably larger and more numerous at 100 mM compared to 500 mM KCl, reflected by lower turbidity at the latter salt concentration (Fig. 3b, c). Condensates formed with FZ95 and FZ100 at different KCl concentrations did not show any significant variation in size, as indicated by confocal analysis: the number of condensates and the associated turbidity were only slightly lower or even slightly higher at 500 mM KCl, for FZ95 and FZ100, respectively (Fig. 3b, c). At these different salt concentrations and upon 30 min incubation, condensates remained round without the compounds and with FZ95, whereas with FZ100 they were mostly irregular (Fig. 3b), consistent with what was observed at 300 mM KCl (Fig. 3a).

An interesting feature of FtsZ-SlmA-SBS condensates is that they are reversible [25] and, accordingly, a shift to higher KCl concentrations induced fast dissociation resulting in lower turbidity and fewer observable condensates in the images (Fig. 3b, c). Control of dilution effects showed a minor turbidity decrease at the maximum volume added (Fig. S2d), well below the turbidity decrease detected upon KCl addition. Turbidity of condensates formed with FZ100 at 100 mM KCl was basically maintained upon salt increase up to 500 mM, and images showed a comparable number of structures with similar shape and size (Figs. 3b, c and S2d). With FZ95, dissociation of condensates formed at 100 mM KCl and shifted to 500 mM slightly reduced the turbidity signal, accompanied with a somewhat lower apparent number of condensates in the images (Figs. 3b, c and S2d). These results suggest that alterations in the response of FtsZ-SlmA-SBS condensates to KCl occur principally with FZ100, but also with FZ95, at times at which the effects of this compound on condensate shape were still not detectable.

### 2.3. BDOBs can dramatically alter the interconversion between FtsZ-SlmA-SBS biomolecular condensates and polymers

**Fig. 4.**
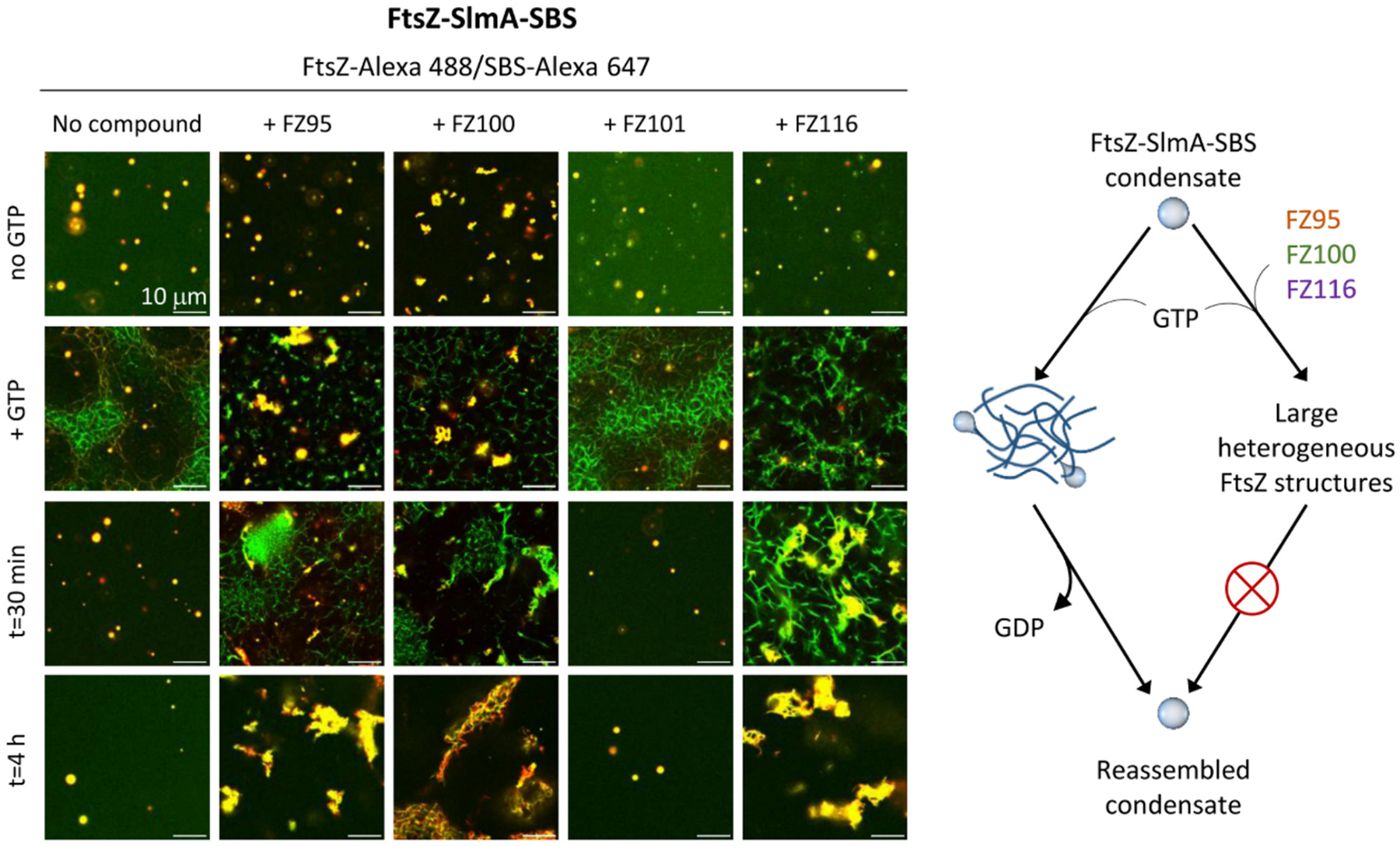
The interconversion between FtsZ-SlmA-SBS condensates and polymers is affected by BDOBs FZ95, FZ100 and FZ116. Confocal images of FtsZ-SlmA-SBS condensates before and after the addition of GTP, showing the formation of GTP-triggered polymers and their evolution with time, with or without compound. Images correspond to the merge of the green and red channels in Fig. S3. All experiments were performed with 10 μM FtsZ, 5 μM SlmA, 1 μM SBS and labeled components and, when present, 20 μM compound and 0.5 mM GTP, in *crowding conditions* (see Materials and Methods) with 150 g/L dextran. A scheme illustrating the effect of the compounds on the polymer/condensate interconversion is shown on the right.

One of the most notable features of FtsZ-SlmA-SBS condensates is their ability to interconvert with FtsZ polymers, in response to GTP addition and depletion due to hydrolysis by FtsZ [25]. Accordingly, in the absence of BDOBs, GTP readily induced the disassembly of most of the FtsZ-SlmA-SBS condensates formed in dextran, with FtsZ-Alexa 488 and SBS-Alexa 647 as tracers, and concurrent formation of FtsZ polymers decorated by SlmA-SBS (Figs. 4 and S3). Polymers were thinner and less defined than the typical FtsZ bundles observed in crowding conditions, because of the antagonistic action of the SlmA nucleoprotein complexes [25]. GTP exhaustion resulted in FtsZ depolymerization, reassembled condensates being the major species after 30 min (Fig. 4).

To determine if BDOBs could modify this behavior, we added GTP to FtsZ-SlmA-SBS condensates with FZ95, FZ100 or FZ116 and, in all cases, we observed the immediate assembly of FtsZ polymers (Figs. 4 and S3). However, polymers were accompanied by a certain number of irregular structures shortly after GTP addition, particularly with FZ95 and FZ100, which tended to cluster as time went by. Importantly, FtsZ-SlmA-SBS condensates were not reassembled in the presence of these compounds even after 4 h, at which time either aggregates or polymers remained visible in the samples (Fig. 4). FZ101 did not modify the normal response of the condensates to GTP addition/depletion (Figs. 4 and S3). We conducted analogous experiments with FZ95 and FZ100 using Ficoll 70 as crowder, rendering equivalent results. Thus, in contrast to the normal interconversion between condensates and polymers, the addition of these BDOBs promoted long-term persistence of polymers and aggregates without condensate reassembly (Fig. S4).

**Fig. 5.**
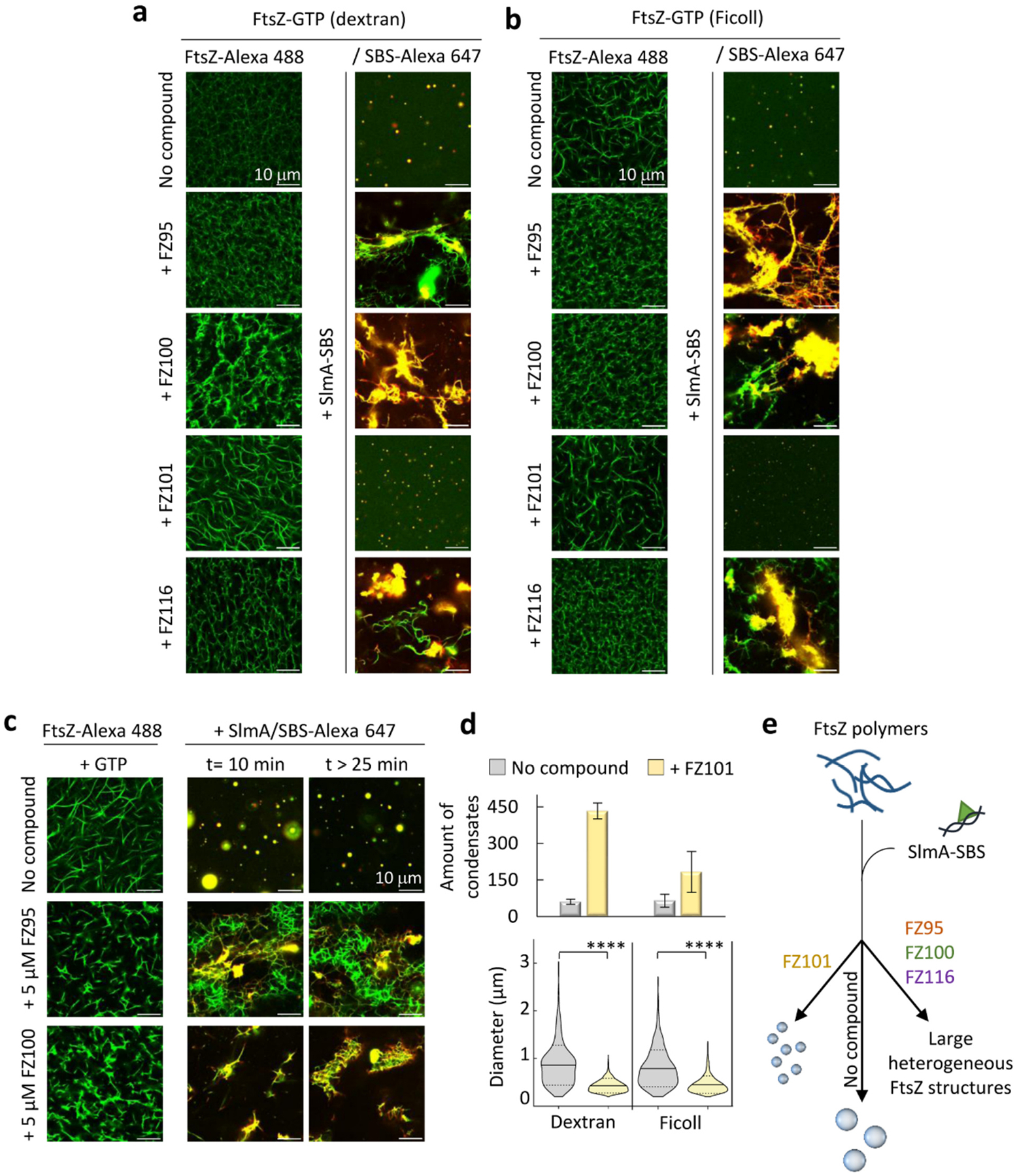
BDOBs FZ95, FZ100 and FZ116 disrupt the assembly of FtsZ-SlmA-SBS condensates from FtsZ polymers. a, b, c) Confocal images of GTP-induced FtsZ polymers in *crowding conditions* (see Materials and Methods) with 150 g/L dextran (a, c) or 200 g/L Ficoll (b), without and with 20 μM (a, b) or 5 μM (c) compounds, before and after addition of SlmA-SBS. Concentrations were 10 μM (a, b) or 5 μM (c) FtsZ, 5 μM (a, b) or 2 μM (c) SlmA and 1 μM (a, b) or 0.4 μM (c) SBS and labeled components. Polymerization of FtsZ was triggered by adding 1 mM GTP. d) Number (± SD) and diameter of condensates in samples from (a, b) without and with FZ101. 6 images (82 × 82 μm fields) from at least two different experiments were analyzed. For dextran, *n* = 354 and 2598 for no compound and FZ101, respectively. For Ficoll, *n* = 389 and 981 for no compound and FZ101, respectively. ****p ≤ 0.0001 by Mann-Whitney Test. Solid line: mean, dashed lines: SD. Error bars correspond to SD. e) Illustration of the structures formed upon addition of SlmA-SBS to FtsZ-GTP polymers.

To further test the effect of the compounds on the interchange between polymers and condensates, we added the inhibitory nucleoprotein complex, SlmA-SBS, to FtsZ polymers in the absence and presence of BDOBs, and obtained similar results with either dextran or Ficoll as crowders (Fig. 5a, b). Without SlmA-SBS, typical FtsZ bundles were observed with or without the compounds, in agreement with previous work under similar conditions [43]. It is worth mentioning that, given the heterogeneous nature of FtsZ polymers under crowding conditions and their time-dependent dissociation, a certain variability in their appearance within the same sample was always found both in the absence and presence of the compounds. Addition of SlmA-SBS to the FtsZ filaments induced the formation of large polymeric arrangements interspersed with irregular structures in the presence of FZ95, FZ100 and FZ116, instead of the rapid formation of condensates observed in their absence (Fig. 5a, b and [25]). These effects were sustained at lower protein and compound concentrations for FZ95 and FZ100 (Fig. 5c). Interestingly, with FZ101, SlmA-SBS induced the appearance of very numerous condensates of size substantially smaller than those formed without compounds (Fig. 5a, b, d). This could be in line with the mild inhibitory effect previously described for FZ101, detected only under crowding conditions [43]. Collectively, these experiments showed that FZ95, FZ100 and FZ116 significantly altered the reversible switching between FtsZ-SlmA-SBS polymers and condensates, blocking disassembly of GTP-induced FtsZ-SlmA-SBS structures and subsequent formation of condensates (Fig. 5e).

## 3. Discussion

With this work, we show notable alterations in the SlmA-dependent FtsZ depolymerization and in the FtsZ-SlmA-SBS condensate-polymer switch induced by BDOB compounds. These compounds were engineered to include an ethylenoxy linker known to greatly increase their antimicrobial activity [39] and were the most potent BDOBs in inhibiting cell division of Gram-positive species such as *S. aureus* and *B. subtilis* [42]. Among the derivatives tested, FZ95 and FZ100 and its *erythro* hydroxy derivative FZ116 promote the hyper-stabilization of *E. coli* FtsZ polymers, even in the presence of a key cytoplasmic FtsZ polymer antagonist, SlmA-SBS. The dominant effect of these derivatives is consistent with their high potency in inhibiting cell division of AcrAB efflux pump-defective *E. coli* [43].

In *E. coli* cells, SlmA-SBS inhibits FtsZ polymerization and Z-ring assembly, thus safeguarding the bacterial chromosome against scission by the division machinery. Our results specifically show that FZ95, FZ100 and FZ116 substantially reduce the susceptibility of FtsZ polymers to disassembly by SlmA-SBS. One potential explanation for these observations is that the compounds directly interfere with FtsZ/SlmA-SBS interactions resulting, for example, from overlap between the unknown SlmA binding region in the FtsZ globular domain [24] and the IDC where these compounds bind [12]. However, we did not find major modification of the FtsZ/SlmA-SBS interactions in the binding experiments conducted, suggesting this may not be the main source of protection. Another possibility is that binding of these small molecules to FtsZ polymers may induce structural or conformational changes, directly or indirectly conferring protection against the SlmA-SBS antagonist. Such changes may be consistent with the previously described hyper-stabilization, reduction of the GTPase activity and slight size modification of the polymers caused by the compounds [43]. Similar stabilization of polymers was observed with the benzamide PC190723 for *H. pylori* and *S. aureus* FtsZ [47] and, together with a reduction in the GTPase activity, with its analogue 8j for *B. subtilis* FtsZ [48].

Interestingly, stabilization of FtsZ polymers by proteins such as ZapA is required for successful Z-ring formation, but this functional stabilization seems to be very different from the one induced by BDOBs. Thus, although both these chemicals and ZapA delay disassembly and reduce FtsZ GTPase activity, ZapA does so by bundling FtsZ filaments whereas BDOBs do not appreciably promote lateral interactions of FtsZ filaments *in vitro*, at least in dilute solution [43]. Moreover, SlmA-SBS seems to efficiently counteract polymer stabilization by ZapA, even at high ZapA: SlmA-SBS ratios [49], but not the stabilization induced by BDOBs. We previously suggested that some features of FtsZ filaments modified by these compounds might be reminiscent of those found in polymers triggered by the slowly hydrolysable nucleotide GMPCPP [43]. Curiously enough, SlmA-SBS also fails to effectively disassemble these polymers, instead inducing higher order arrangements [22,24]. This suggests that non-physiological factors slowing down GTP hydrolysis, such as BDOBs, may interfere with FtsZ polymers regulation by SlmA-SBS, potentially compromising nucleoid integrity and cell viability.

**Fig. 6.**
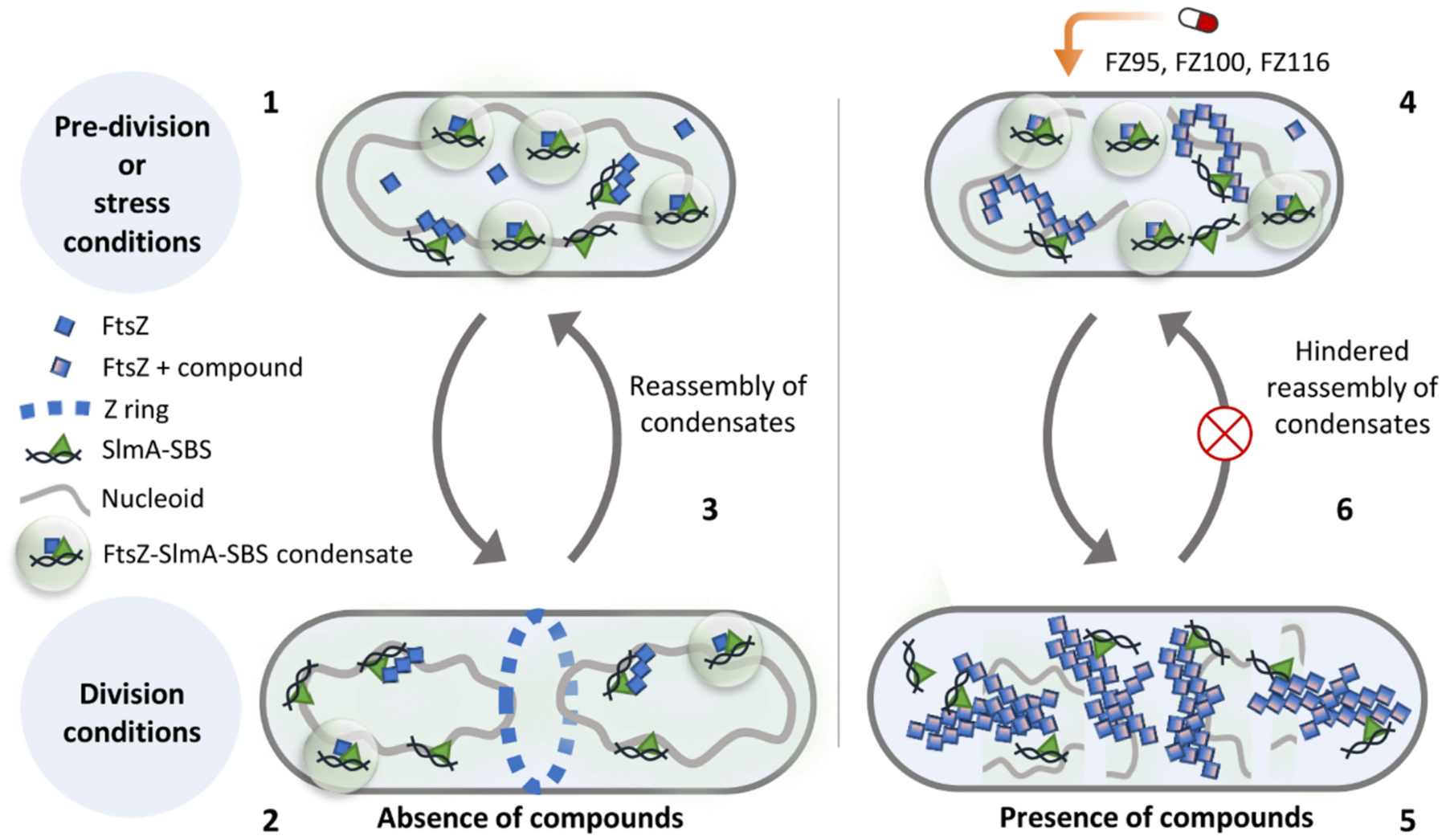
Scheme of the potential effects of certain BDOBs on the interplay between FtsZ and the inhibitory SlmA-SBS system. 1) Under non-division conditions, SlmA-SBS complexes antagonize Z-ring assembly in the vicinity of the nucleoid to protect this crucial structure. FtsZ-SlmA-SBS would form biomolecular condensates that effectively sequester FtsZ, thus helping to hinder Z-ring assembly. Under stress conditions, the chances of survival increase through the emergence of persister cells with arrested division, possibly facilitated by the assembly of FtsZ-SlmA-SBS biomolecular condensates at low GTP levels. 2) Under division conditions, the Z-ring is formed at the cell center where the Ter-region of the duplicated chromosomes lacks SBS sites. SlmA bound to SBS sequences outside the Ter-region inhibits Z-ring assembly by the FtsZ outside midcell, avoiding damage to the segregated nucleoids. Increasing GTP levels upon stress release would facilitate disassembly of condensates, FtsZ polymerization and division resumption. 3) In case of recurrent stress cycles, decrease of GTP levels would facilitate biomolecular condensate reassembly and quiescence. 4) BDOBs with potential antimicrobial activity against *E. coli* hyperstabilize FtsZ polymers, rendering them less sensitive to SlmA-SBS inhibition. Failure of these mechanisms may ultimately cause damage to the nucleoid under non-division conditions. 5) Under division conditions, the presence of the compounds would lead to aberrant FtsZ-SlmA-SBS structures in the crowded cytoplasm, hindering the formation of the Z-ring and 6) inhibiting reassembly back into condensates. This would potentially compromise the cell division process itself, along with nucleoid integrity and stress sensing mechanisms.

The inhibitory effect of these compounds, tested by reconstitution of the FtsZ-SlmA nucleoprotein complex in cell-like conditions, seems to be mainly focused on the dynamic switch between FtsZ bundles and FtsZ-SlmA-SBS condensates, rather than affecting the ability of FtsZ within the condensates to polymerize or the assembly of condensates in the absence of GTP. Nonetheless, the observed reduction in the ability to switch between polymerized and condensate forms could translate into a lower capacity to respond to environmental changes (Fig. 6). The BDOBs-induced stabilization of FtsZ polymers against dissociation by SlmA-SBS is in agreement with previous *in vitro* studies showing a similar stabilization effect on FtsZ filaments and bundles, together with a reduction in their GTPase activity [43]. However in this case, unlike with FtsZ polymers on their own, the presence of SlmA-SBS promotes, under these crowding conditions, the appearance of additional large structures that seem to be irreversible. These findings suggest that these compounds are active against the hetero-associations of FtsZ with other proteins. Such associations remain essentially unexplored as drug targets, even though most of the available small molecules acting on cell division are directed against FtsZ.

Because of their implications in the regulation of essential cell cycle processes, biomolecular condensates have been suggested as innovative targets for the development of strategies to curb bacterial infections [20,33,34]. Connections between these membraneless compartments and the emergence of persister cells tolerant to antimicrobials suggest that approaches based in disrupting phase separation would be less likely to be circumvented by resistance mechanisms than others, particularly those relying on modifications of individual proteins. Emerging evidence shows that phase separation needs to be considered to explain the killing action of some antimicrobial peptides [35,36]. Our results strongly suggest that this might be also the case of BDOBs, given the disruption they induce in the interconversion between FtsZ biomolecular condensates and polymers, which are finely tuned in response to GTP concentration. This alteration may be detrimental for the inhibition mechanisms mediated by SlmA under normal growth conditions and compromise their potential role in dormancy (Fig. 6). Through the alarmone (p)ppGpp produced under stress [50], a decrease in GTP levels [51] would favor sequestration of FtsZ in FtsZ-SlmA-SBS biomolecular condensates, resulting in transient cell division arrest. This would be consistent with subcellular structures enriched in FtsZ previously reported in dormant cells [52]. Once the stress is over, replenished GTP levels would trigger FtsZ polymers emerging from the condensates to engage into Z-ring formation and normal division. By profoundly altering these mechanisms, BDOBs may reduce the chances of the cells to survive in different kinds of stresses and perhaps the possibility of developing resistance.

We provide evidence that, in addition to modifying FtsZ polymerization features or perhaps as a result of these modifications, BDOBs can substantially alter the regulation of *E. coli* FtsZ polymers by the antagonistic nucleoid occlusion factor SlmA. It would be interesting to determine whether the other negative spatial regulator from *E. coli,* the Min system, may also fail to efficiently disassemble BDOBs-bound FtsZ polymers. Although nucleoid occlusion and Min system mechanisms share some features, there are important differences suggesting that modification of the FtsZ polymers by BDOBs might have a distinct repercussion on the Min system inhibition. In addition, it would be useful to understand how polymers modified by BDOBs respond to positive regulators and if analogous mechanisms operate in the variety of bacterial species on which these compounds display activity. We also show data suggesting that modification of the phase separation behavior of division proteins might improve BDOBs efficacy as antimicrobial leads. More research is needed to dissect the potential of biomolecular condensates involving essential cell machineries as new targets to fight antimicrobial resistance. Nevertheless, the experimental data here presented provide a solid and promising starting point to support the feasibility and relevance of the project.

## 4. Materials and methods

### 4.1. Materials and working conditions

Salts, buffer components of analytical grade and nucleotides (GTP) were purchased from Sigma. Ficoll 70 and dextran 500, from GE Healthcare and Sigma respectively, were dialyzed before being used in 50 mM Tris-HCl, pH 7.5, 300 mM KCl. Final concentrations of the stocks solutions were calculated from their refractive index, using refractive index increments (dn/dc) of 0.141 mL/g for Ficoll 70 [53] and 0.142 mL/g for dextran 500 [54]. BDOBs (FZ95, FZ100, FZ101 and FZ116) were synthesized as described [38,43], and stocks were prepared by dissolving the compounds in DMSO. Final concentration of DMSO in the experiments was kept within the range 0.5–2 %, as specified. Experiments were conducted in *crowding conditions* (50 mM Tris-HCl, pH 7.5, 300 mM KCl, 1 mM MgCl_2_ with dextran or Ficoll at the specified concentrations) or in *dilute solution buffer* (50 mM Tris-HCl, pH 7.5, 300 mM KCl, 5 mM MgCl_2_). An enzymatic GTP regeneration system (RS; 2 units/mL acetate kinase and 15 mM acetyl phosphate, both from Merck) was added when specified to keep the FtsZ polymers assembled during sufficient time to be studied [55].

### 4.2. E. coli FtsZ and SlmA proteins purification, labeling and DNA hybridization

SlmA was purified as described elsewhere [22,23] and dialyzed before use in 50 mM Tris-HCl, pH 7.5, 300 mM KCl. FtsZ was purified following a previously reported protocol (calcium induced precipitation method, [56]) and dialyzed before use in 50 mM Tris-HCl, pH 7.5, 300 mM KCl, 5 mM MgCl_2_.

FtsZ and SlmA were covalently labeled at the amino groups with Alexa Fluor 488 succinimidyl ester dye (Thermo Fisher Scientific) as reported earlier [22,45]. Labeling ratios for both proteins were determined to be 0.4–0.5 mol of dye per mole of protein, as calculated from the molar absorption coefficients of the proteins and the dye. Labeled proteins were stored in aliquots at −80 °C until used.

Double-stranded DNA with the consensus SlmA binding sequence (SBS; 5’-AAGTAAGTGAGCGCTCACTTACGT-3′; bases recognized by SlmA underlined) was obtained by hybridization of complementary oligonucleotides (HPLC-grade; IDT), either unlabeled or labeled with Alexa Fluor 647, with Alexa Fluor 488 or with fluorescein at its 5′ end [22].

### 4.3. Fluorescence anisotropy

Fluorescence anisotropy experiments were performed in a Spark^®^ Multimode microplate reader (Tecan) at 25 °C, using excitation and emission filters at 485 nm and 535 nm, respectively, as described in [49]. Samples were loaded in 384-well, black polystyrene, flat bottom microplates (Corning).

FtsZ depolymerization was monitored by following the time-dependent anisotropy of samples containing 50 nM FtsZ-Alexa 488 (as tracer) and unlabeled FtsZ up to 10 μM, after triggering polymerization with GTP. Samples contained different concentrations of SlmA and SBS, in the absence or presence of various concentrations of the compounds (1 % DMSO final concentration). Control experiments showed DMSO does not contribute to the effects induced by the compounds (Fig. S5a), and ruled out effects of SBS in the absence of SlmA (Fig. S1f). No changes in the total intensity measured were observed in the presence of the compounds, and background contributions were always subtracted. Curves shown in the figures are representative of, at least, 3 independent replicates. Areas under the anisotropy curves were determined using trapezoids for integration, equivalent to estimations using SigmaPlot software, in order to facilitate comparison between different conditions. The values correspond to the average of at least three independent measurements, as specified, and were normalized to those obtained for FtsZ without any compound or SlmA-SBS. Errors were determined by error propagation from SD. Depolymerization times, estimated from the time point at which no change in the anisotropy signal (full disassembly) is observed, were also normalized with respect to the sample in the absence of compound or SlmA-SBS.

Binding experiments were performed by measuring fluorescence anisotropy from samples containing SlmA (1 μM), SBS-Fl (0.01 μM) and increasing concentrations of FtsZ, in the absence or presence of compounds (20 μM), right after the addition of 2 mM GTP to trigger polymerization. The shift of anisotropy (ΔAnisotropy) was calculated by subtracting the value of anisotropy from the samples without FtsZ. Values are the average of 4 independent replicates. Isotherm without compound was fitted using a 1:1 binding model compatible with the data employing BIOEQS software [57,58], to obtain apparent binding parameters. Uncertainty was obtained by rigorous confidence limit testing at the 67 % using the same software.

### 4.4. Fluorescence correlation spectroscopy

FCS assays were performed using a Microtime 200 (Picoquant) time-resolved confocal fluorescence microscope equipped with a pulsed laser diode head (LDH-P-C-485) for excitation, as described in [49]. Samples included 10 μM FtsZ (10 nM FtsZ-Alexa 488 as tracer), 2 μM SlmA, 0.4 μM SBS and 2 mM GTP, with RS (see above). Experiments were also conducted with SBS (0.4 μM, 10 nM SBS-Alexa 488 as tracer), SlmA (2 μM), FtsZ (10 μM) and 2 mM GTP, with RS. When required, compounds were added at 20 μM (0.5 % DMSO final concentration). Samples were loaded in chambers treated by PEGylation, which prevents nonspecific adsorption of proteins to the surface.

In each experiment, 5 autocorrelation curves were measured and analyzed as described somewhere else [49] using FFS Data Processor software (version 2.4 extended, Scientific Software Technologies Center, Belarus [59]) with models that include a term for the triplet state dynamics. Profiles corresponding to samples with FtsZ-GTP were analyzed using a two-diffusing species model, fixing the fastest one to the diffusion value obtained for FtsZ in the absence of GTP. Profiles recorded in the presence of SlmA-SBS, with and without the compounds, could be also described by this model and <*D*_*app*_> values were calculated from the two diffusion coefficients and their fractional contributions to the autocorrelation profiles, allowing to compare the overall species diffusion between different conditions. Profiles with only SBS-Alexa 488 with or without SlmA were fitted with a model involving a single diffusion component. Diffusion values are the average of, at least, 3 independent replicates ± SD. DMSO controls showed no contribution to the profiles (Fig. S5b, c).

### 4.5. Dynamic light scattering

DLS experiments were conducted in a Protein Solutions DynaPro MS/X instrument as earlier described [46]. Samples contained 10 μM FtsZ, 2 μM SlmA and 0.4 μM SBS. If present, compounds were added at 20 μM (0.5 % DMSO final concentration). Assembly of FtsZ polymers was triggered by the addition of 2 mM GTP, including the GTP RS (see above).

Autocorrelation profiles were exported using Dynamics V6 software and analyzed using user-written scripts and functions in MATLAB (version 7.10, Mathworks, Natick, MA) as described elsewhere [46] to get the *D*_*app*_ values and the uncertainties, estimated *via* least-squares modeling using a variation of the sum-of-squares profile method described by Saroff [60]. Each independent replicate is the average of 11–40 acquisitions. Diffusion coefficients reported for each sample are the values obtained by simultaneous fit of 2–4 independent replicates ± SD representing confidence limits at 68 %. Similar results were obtained from the average of the diffusions from individual analysis of the replicates ± SD calculated by error propagation. Controls discarded a contribution coming from DMSO (Fig. S5c).

### 4.6. Preparation of condensates in crowding conditions

Solutions were prepared by mixing the proteins and SBS, with or without the compounds, in *crowding conditions* containing either 200 g/L Ficoll or 150 g/L dextran. Unless otherwise stated, samples with condensates were measured or visualized after 30 min incubation at room temperature, except for salt shift experiments where measurement or visualization started immediately after addition of KCl. When required, GTP was directly added to trigger FtsZ polymerization.

### 4.7. Turbidity measurements

Turbidity of FtsZ-SlmA-SBS condensates was monitored in a Varioskan Flash plate reader (Thermo Fisher Scientific, MA, USA). Absorbance at 350 nm was measured after incubation of the samples for 30 min, as previously described [61]. Samples, prepared at room temperature in *crowding conditions* with dextran at different KCl concentration, as specified, contained 10 μM FtsZ, 5 μM SlmA and 1 μM SBS, with or without 20 μM compound (0.5 % DMSO final concentration). DMSO controls showed no contribution (Fig. S5d). Salt shift experiments were conducted by adding KCl in two sequential steps on the sample in 50 mM Tris-HCl, pH 7.5, 100 mM KCl, 1 mM MgCl_2_ with 150 g/L dextran, incubated during 30 min. Absorbance was measured during 30 min immediately after each KCl addition, with no significant variation. Figures show the average of the specified values (*n* ≥ 3) ± SD.

### 4.8. Confocal microscopy

Image acquisition was performed in a Leica TCS-SP5 inverted confocal microscope, equipped with a HCX PL APO 63× oil immersion objective (N.A. 1.4, Leica, Mannheim, Germany). 488 and 633 nm laser lines were used to excite Alexa Fluor 488 and Alexa Fluor 647 dyes, respectively. Samples were observed in silicon chambers glued to a glass coverslip. DMSO coming from 20 μM compounds was 0.5–1 %, with no contribution neither in the condensates nor the polymers (Fig. S6a, b). Images with only SlmA-SBS showed lack of detectable effects induced by the compounds (Fig. S6c). Controls with SlmA as the labeled element (confocal and differential interference contrast images, DIC images) or with unlabeled elements (DIC images) showed a response of the FtsZ-SlmA-SBS condensates with the compounds to GTP addition similar to the above described effects for condensates containing labeled FtsZ or SBS as tracers (*cf*. Figs. 4, second row, and S6d). No effects derived from the presence of DMSO were found when SlmA-SBS was added to samples containing FtsZ-GTP and DMSO (*cf*. Fig. 5a, first row and S6e). Additional controls discarded modifications in the behavior of FtsZ, with or without the compounds, induced by SBS when SlmA was not present (Fig. S7), in contrast to the effects reported in Figs. 4 and 5, from samples containing SlmA-SBS. ImageJ [62] was used to prepare images and to measure the area of the condensates in the images with the particle analysis option, after noise reduction by applying a Kuwahara filter and threshold correction by visual inspection. Only when specified, brightness increase was conducted using Microsoft PowerPoint (correction applied uniformly to the entire image). Several images corresponding to different observation fields were recorded, and reported images are representative of at least three independent experiments, unless otherwise stated. Condensates diameter distribution was represented as violin plots using Origin 2024b and statistical analysis was performed in GraphPad Prism 10.4.1 and Origin 2024b with identical results.

## Supplementary Material

Supplementary file

## Data Availability

All data are available from the corresponding authors upon request.

## Appendix A. Supplementary data

Supplementary data to this article can be found online at https://doi.org/10.1016/j.ijbiomac.2025.148516.
