# Supplementary material for: Benzodioxane-benzamides targeting bacterial cell division protein FtsZ potentially disrupt SlmA-mediated nucleoid occlusion and reversible biomolecular condensation
Source: Int J Biol Macromol. Author manuscript; Available in PMC 2025 Dec 10. (PMC12693692; doi:10.1016/j.ijbiomac.2025.148516)
Supplement: Supplementary file [file NIHMS2127794-supplement-Supplementary_file.pdf]

Supplementary Material

Benzodioxane-benzamides targeting bacterial cell division protein FtsZ potentially disrupt SlmA-mediated nucleoid occlusion and reversible biomolecular condensation

Marta Sobrinos-Sanguino[a], Inés Barros-Medina[a], Lorenzo Suigo[a,b,c], Alessia Lanzini[b], Ermanno Valoti[b], William Margolin[c], Valentina Straniero[b,*], Begoña Monterroso[d,1,*], Silvia Zorrilla[a,1,*]

[a]Department of Cellular and Molecular Biosciences, Centro de Investigaciones Biológicas Margarita Salas, Consejo Superior de Investigaciones Científicas (CSIC), 28040 Madrid, Spain

[b]Dipartimento di Scienze Farmaceutiche, Università degli Studi di Milano, Via Luigi Mangiagalli, 25, 20133 Milano, Italy

[c]Department of Microbiology and Molecular Genetics, McGovern Medical School, University of Texas, Houston 77030, TX, USA

[d]Department of Crystallography and Structural Biology, Instituto de Química Física Blas Cabrera, Consejo Superior de Investigaciones Científicas (CSIC). 28006 Madrid, Spain

[1]Equal contribution in alphabetical order

*Corresponding authors: valentina.straniero@unimi.it (V.S.); bmonterroso@iqf.csic.es (B.M.); silvia@cib.csic.es (S.Z.).

Contents:

Figures S1-S7 with legends

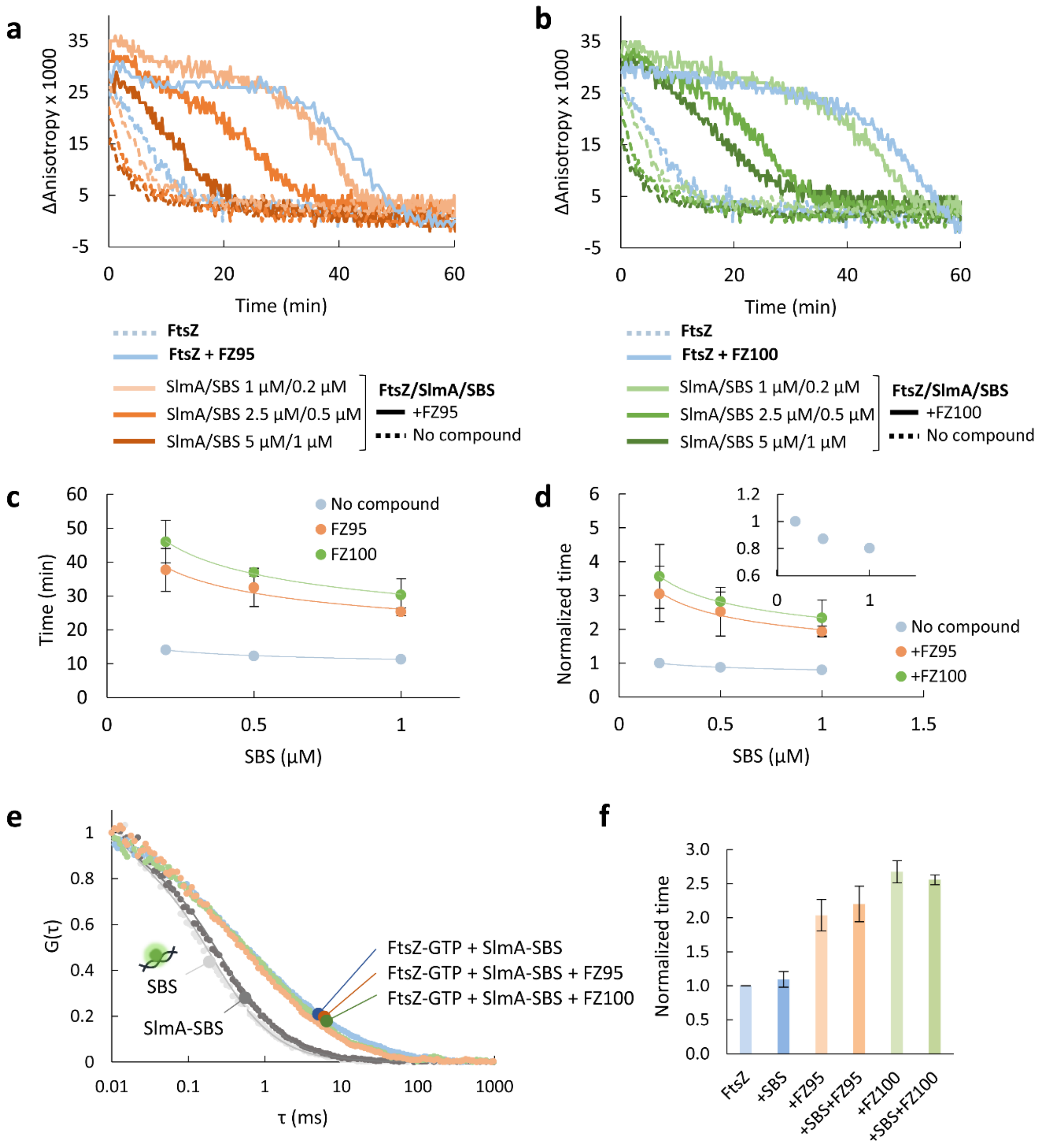

Figure S1. FZ95 and FZ100 counteract SlmA-SBS induced FtsZ polymers disassembly in dilute solution.

a, b) Depolymerization profiles of FtsZ with variable SlmA-SBS, with and without FZ95 (a) or FZ100 (b), monitored by fluorescence anisotropy. The value reached after depolymerization was subtracted. Profiles of FtsZ with and without the compounds are shown for reference. The SlmA:SBS molar ratio was kept at 5:1. Samples were measured right after the addition of 1 mM GTP.

c) Depolymerization times estimated from the anisotropy curves in (a) and (b).

d) Depolymerization times in (c) normalized to that of FtsZ alone. Inset magnifies values for FtsZ-SlmA-SBS without compounds. Errors are within the symbols.

e) Normalized FCS autocorrelation curves of 0.4 μM SBS (10 nM SBS-Alexa 488 as tracer) in the presence of SlmA (2 μM) and FtsZ-GTP (+RS), with or without FZ95 or FZ100. Curves of SBS alone and with SlmA are shown for reference. Solid lines correspond to the fit of the models indicated in Materials and Methods. When present, GTP was at 2 mM.

f) Depolymerization times normalized to that of FtsZ alone in samples where SlmA is not present.

The concentrations of FtsZ and compounds, when present, were 10 μM (with 50 nM FtsZ-Alexa 488 as a tracer in (a-d, f) and 20 μM, respectively. Experiments were performed in *dilute solution buffer* (see Materials and Methods), and are representative of at least three independent replicates (a, b, e) or the average of 3 independent measurements ± SD (c, d, f).

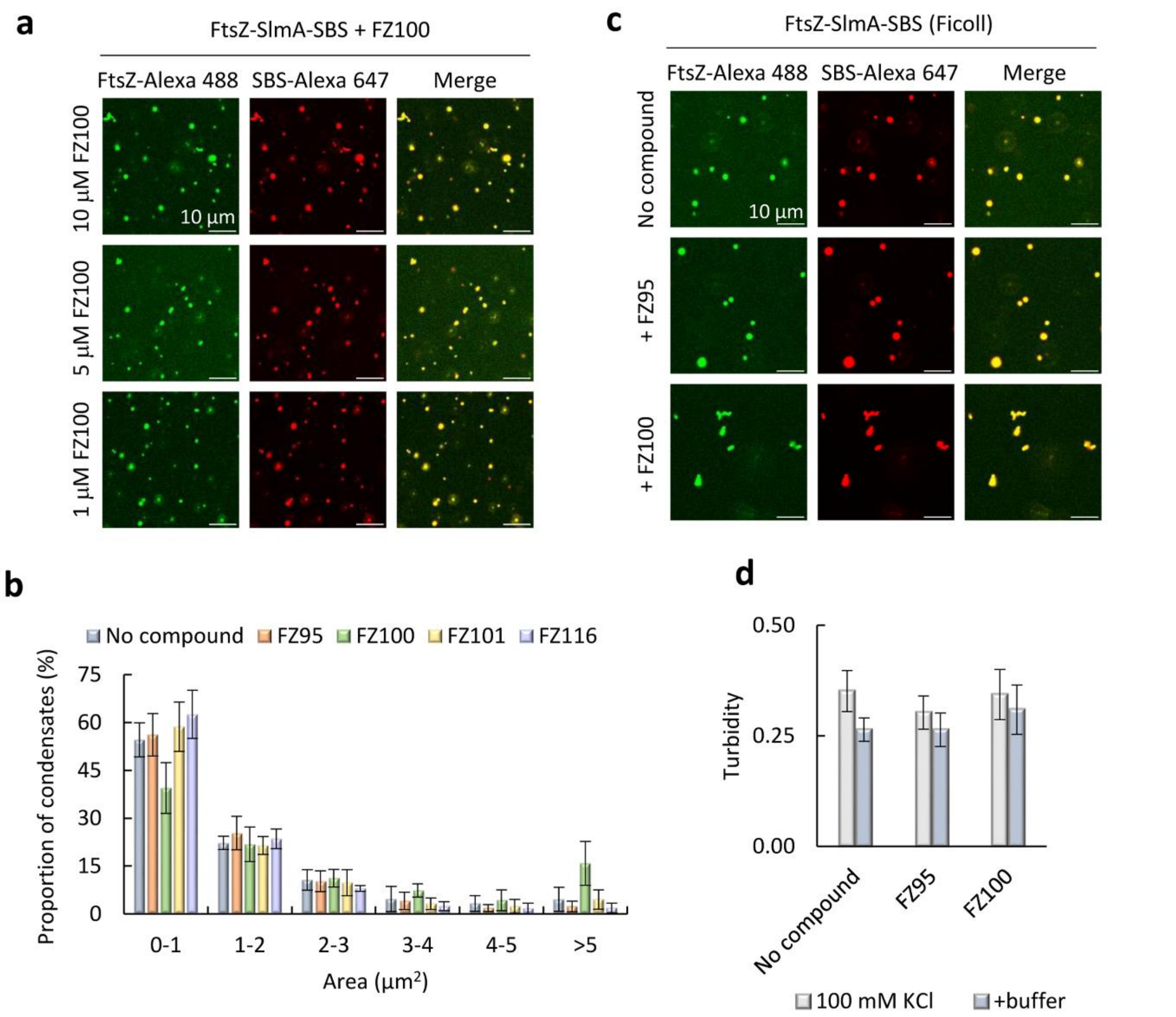

Figure S2. FtsZ-SlmA-SBS condensates in the presence of the compounds, under different conditions.

a) Confocal images of condensates with the specified concentrations of FZ100.

b) Amount of condensates grouped by their area, with or without compounds FZ95, FZ100, FZ116 and FZ101, obtained from analysis of the experiments shown in Figure 3a. Data are the average percentage of condensates within that area interval ± SD.

c) Images showing condensates without and with FZ95 and FZ100 in Ficoll as crowder.

d) Controls to discard major dilution effects in the salt shift experiments. Added buffer (50 mM Tris-HCl, pH 7.5) volume corresponds to that required to achieve the highest target KCl concentrations in the experiments shown in Figure 3c. Reported values are the average of at least three independent measurements ± SD.

For all experiments, concentrations were 10 µM FtsZ, 5 µM SlmA, 1 µM SBS and, when present, labeled components. Compounds were at 20 µM unless otherwise specified. FtsZ-SlmA-SBS condensates were incubated for 30 minutes. Experiments were performed in *crowding conditions* with 150 g/L dextran, except for (c) in which dextran was replaced by 200 g/L Ficoll.

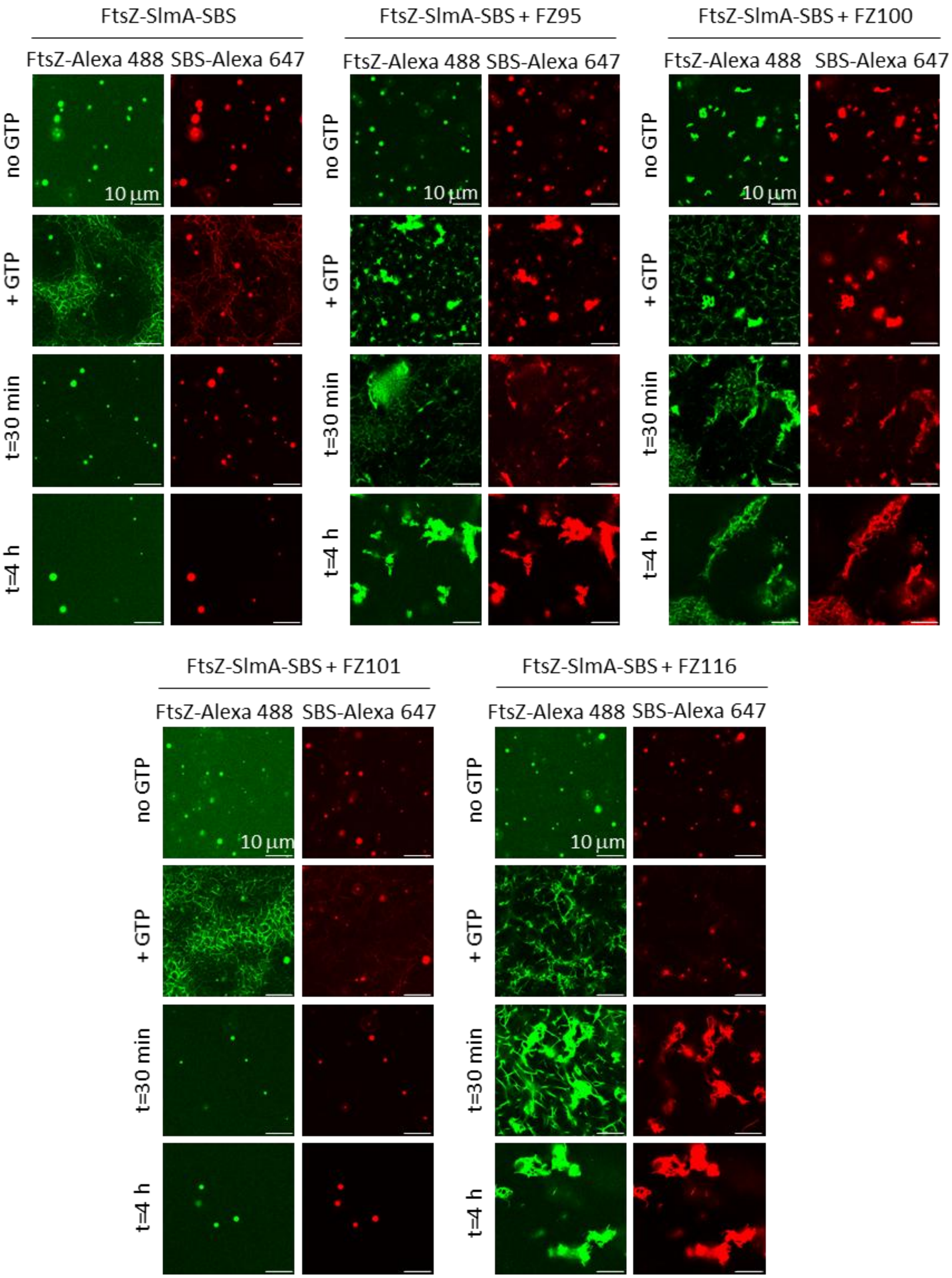

Figure S3. GTP-dependent FtsZ-SlmA-SBS condensates/polymers interconversion is blocked by FZ95, FZ100 and FZ116.

Red and green channels corresponding to merged confocal images shown in Figure 4. All experiments were performed with 10 μM FtsZ, 5 μM SlmA, 1 μM SBS and labeled components and, when present, 20 μM compound and 0.5 mM GTP, in *crowding conditions* with 150 g/L dextran.

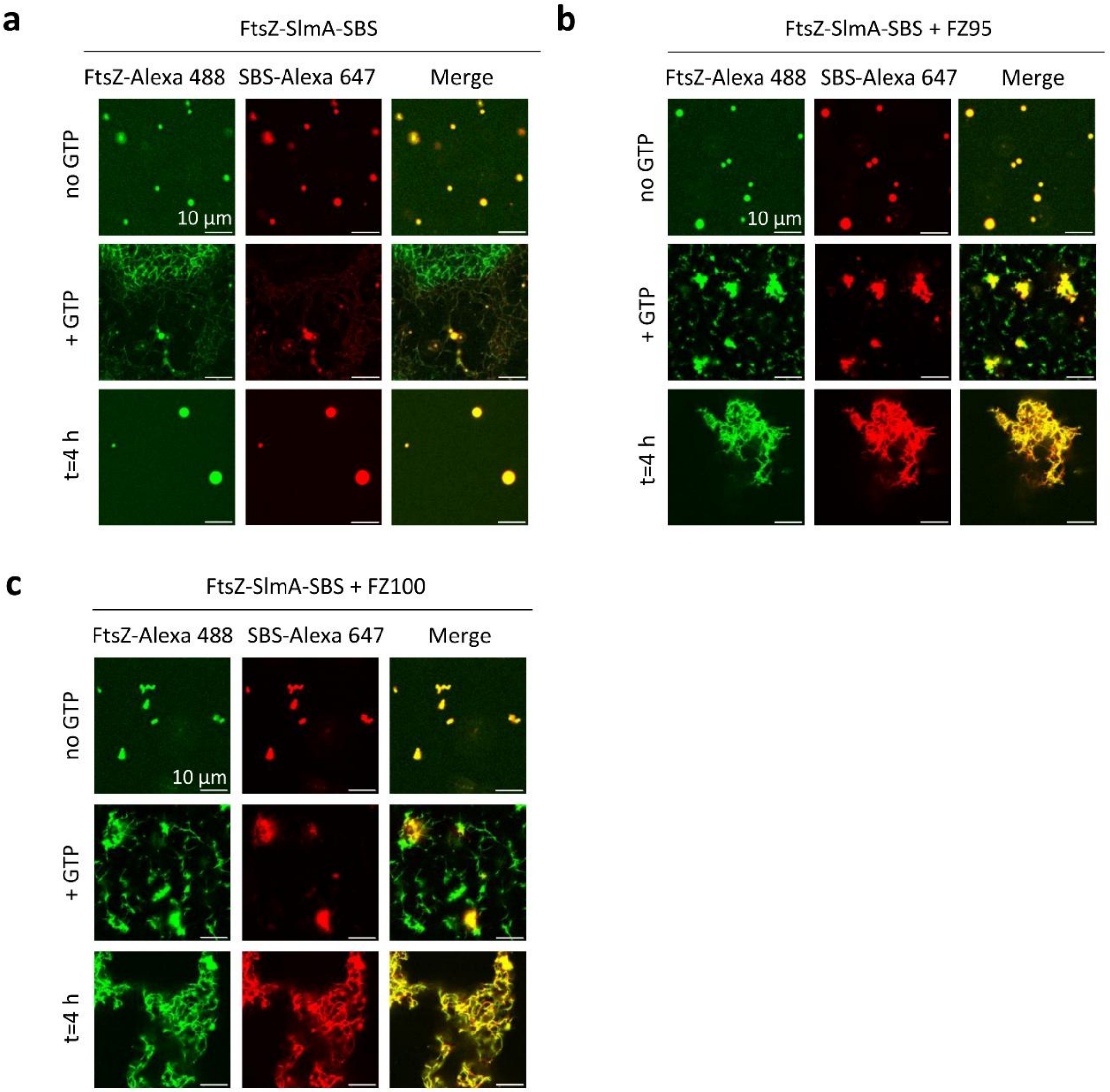

Figure S4. FZ95 and FZ100 perturb FtsZ-SlmA-SBS condensates/polymers interconversion in Ficoll 70 as crowder.

Confocal images of condensates in the absence (a) and presence of FZ95 (b) or FZ100 (c) before, immediately after (+GTP) or 4 hours after triggering polymerization with 0.5 mM GTP. The concentrations of FtsZ, SlmA and SBS and labeled components were 10, 5 and 1 µM, respectively. Compounds were at 20 µM. Experiments in *crowding conditions* with 200 g/L Ficoll.

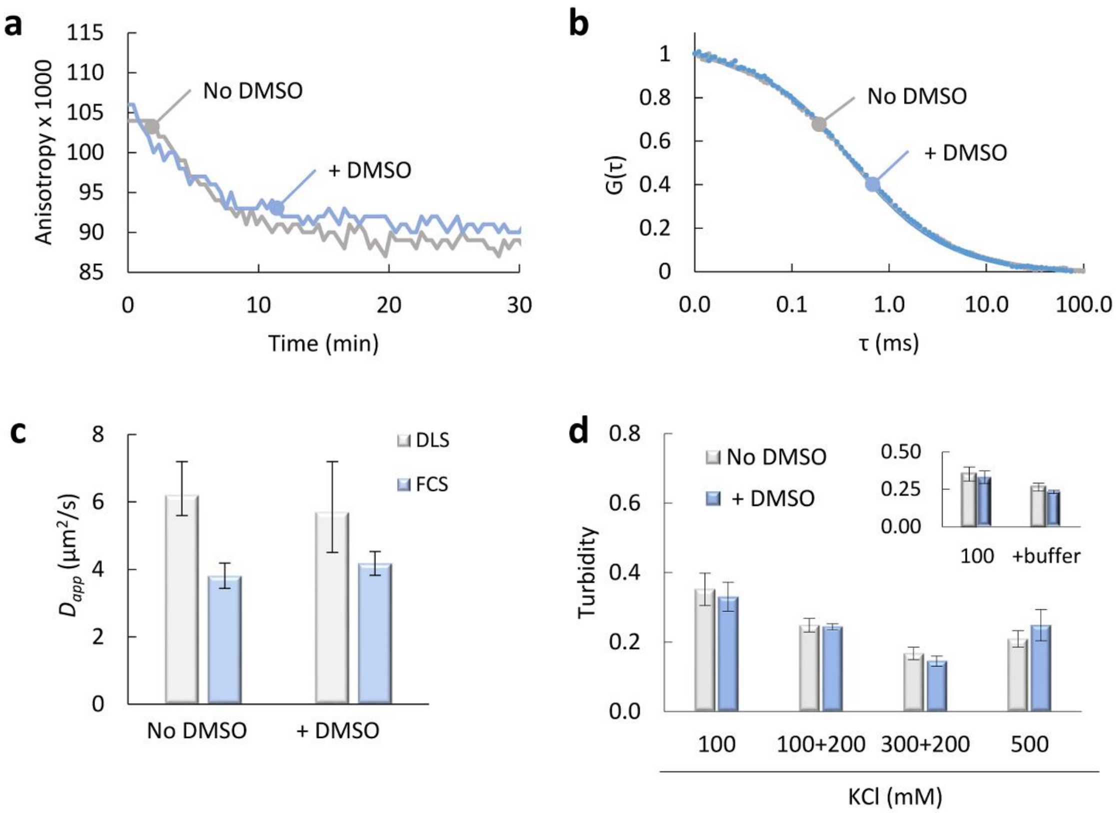

Figure S5. DMSO controls for experiments conducted in crowding or diluted conditions.

a) Fluorescence anisotropy profiles showing disassembly of FtsZ filaments (50 nM FtsZ-Alexa 488), after 1 mM GTP addition, in the presence of SlmA-SBS, with and without 1% DMSO.

b) Normalized FCS curves of FtsZ (with 10 nM FtsZ-Alexa 488 as tracer) in the presence of SlmA-SBS and GTP RS, with and without 2% DMSO.

c) Apparent translational diffusion coefficient (D_{app}) values obtained by DLS and FCS (autocorrelation curves in b) in samples of FtsZ-SlmA-SBS with and without DMSO (0.5% in the case of DLS).

d) Effect of KCl concentration on the turbidity of FtsZ-SlmA-SBS condensates in the absence and presence of 0.5% DMSO. A possible dilution effect (inset) was discarded adding equivalent volumes of 50 mM Tris-HCl, pH 7.5 (i.e., no KCl). Samples in *crowding conditions* with 150 g/L dextran.

SlmA and SBS were at 5 µM and 1 µM respectively (a, d), or at 2 µM and 0.4 µM (b, c), while FtsZ was at 10 µM in all cases. Experiments were performed in *dilute solution buffer* except as specified in (d). In (a, b) curves are representative of at least three independent measurements. FCS values in (c) and turbidity in (d) are the average of at least three experiments ± SD. DLS data in (c) are obtained by simultaneous fit to 2-4 independent replicates ± SD.

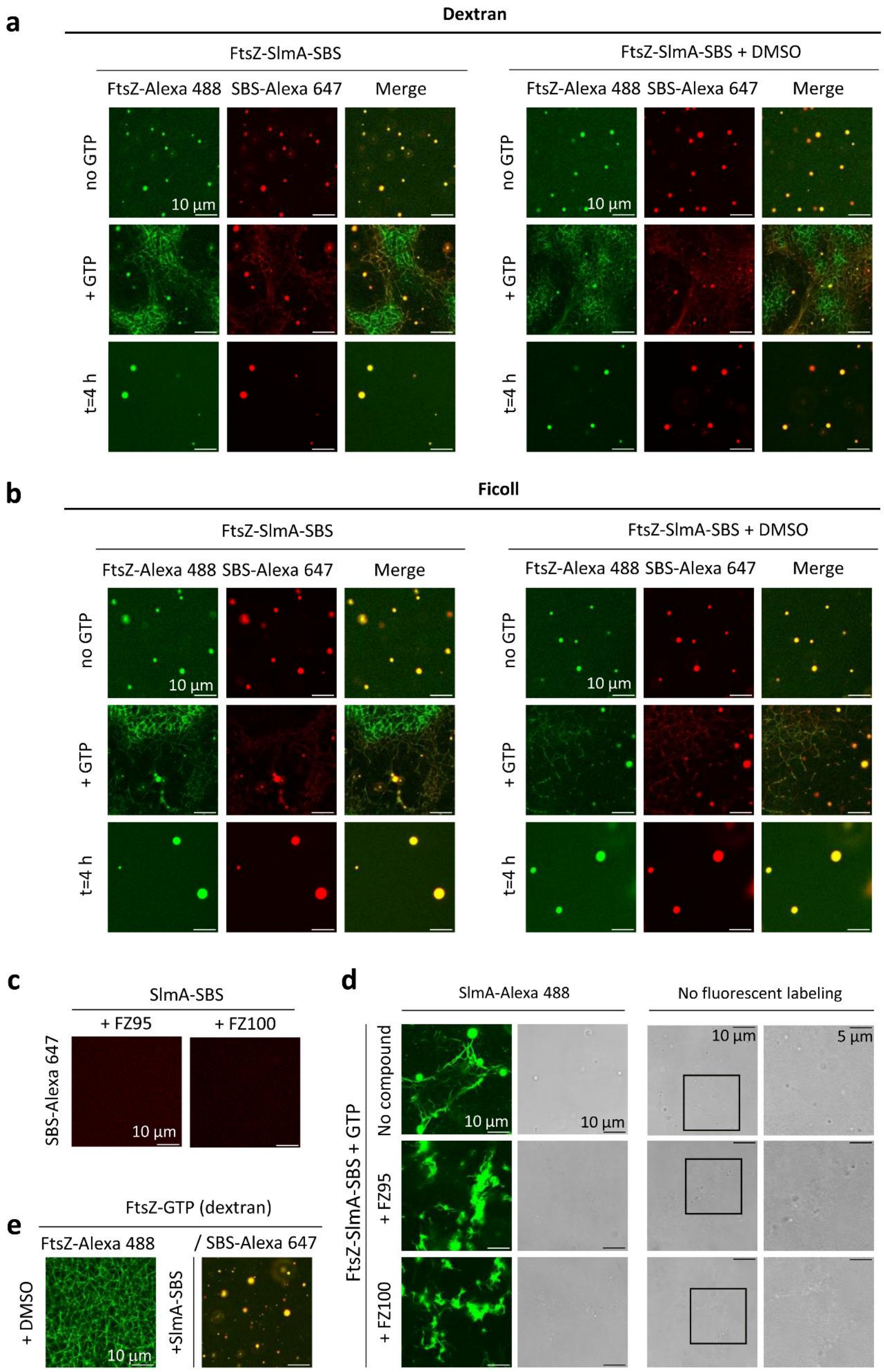
a
Dextran
FtsZ-SlmA-SBS
FtsZ-SlmA-SBS + DMSO
FtsZ-Alexa 488
SBS-Alexa 647
Merge
no GTP
+ GTP
t=4 h
10 µm
b
Ficoll
c
SlmA-SBS
+ FZ95
+ FZ100
SBS-Alexa 647
d
SlmA-Alexa 488
No fluorescent labeling
FtsZ-SlmA-SBS + GTP
No compound
5 µm
e
FtsZ-GTP (dextran)
/ SBS-Alexa 647
+ DMSO
+SlmA-SBS

Figure S6. Controls for confocal microscopy experiments.

a, b) Confocal images of FtsZ-SlmA-SBS samples with 150 g/L dextran (a) or 200 g/L Ficoll (b) showing the evolution without (left) or with 1% DMSO (right). Samples were visualized after 30 min incubation (no GTP), followed by the addition of 0.5 mM GTP (+GTP) and final examination 4 hours later (t=4 h).

c) Images of SlmA-SBS samples in the presence of FZ95 or FZ100, as specified.

d) Representative images of FtsZ-SlmA-SBS samples with or without compounds using SlmA-Alexa 488 as alternative labeling (left), or without any labeled elements (right). After a 30-min incubation, 2 mM GTP was added to the samples, before visualization. On the samples without labeling, image boxes depict the areas further magnified for a better visualization of the structures, on the right. Brightness in the brightfield images was increased by 50% in the case of the samples containing SlmA-Alexa 488, and by 20% in the samples without any fluorescently labeled element.

e) Images of FtsZ-GTP with 0.5% DMSO and addition of SlmA-SBS.

Concentrations were 10 µM FtsZ, 5 µM SlmA, and 1 µM SBS and labeled components. Samples were prepared in *crowding conditions* with 150 g/L dextran unless otherwise stated.

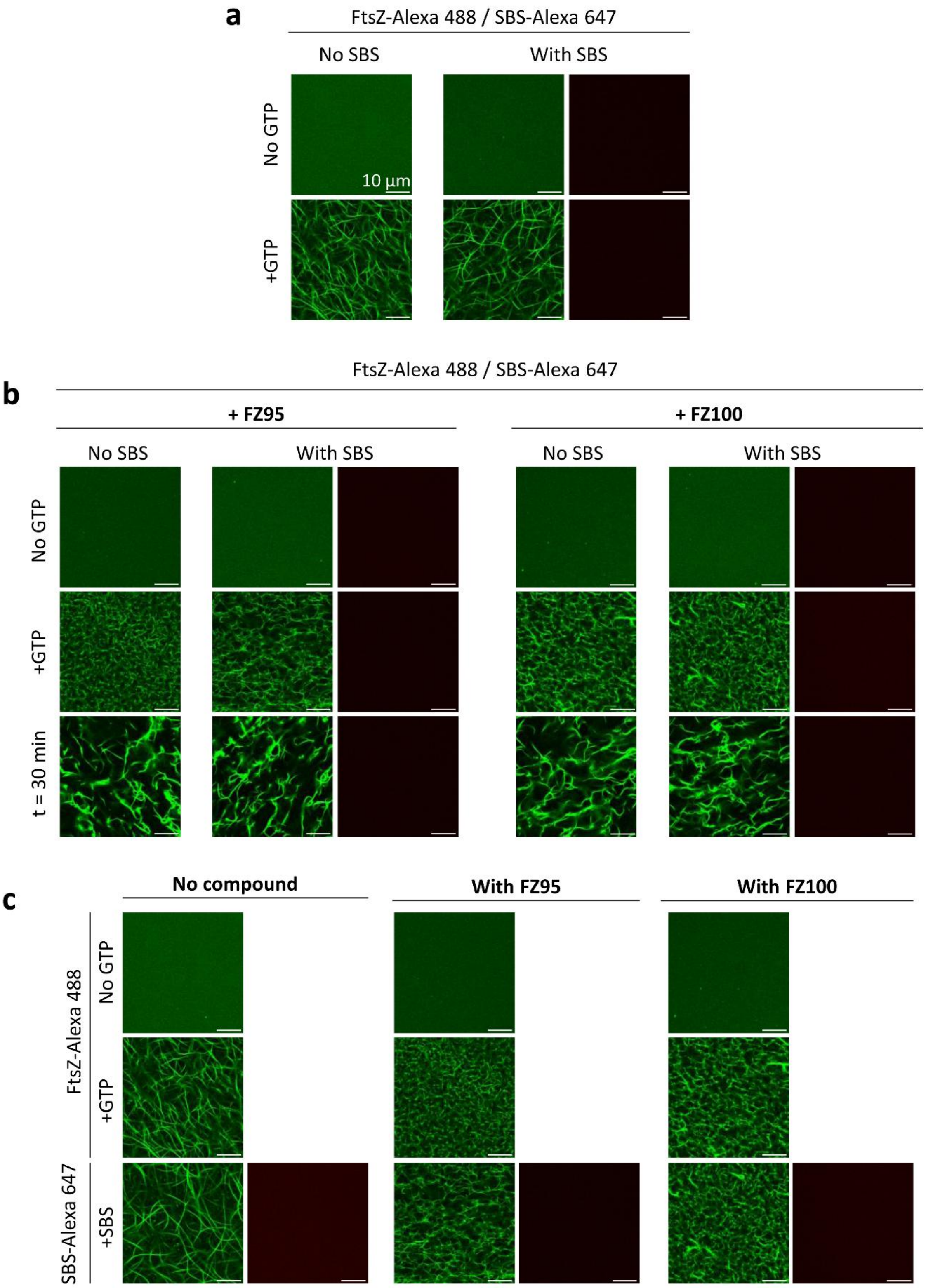

Figure S7. Controls of confocal microscopy experiments with and without SBS in samples that do not contain SlmA.

a) Confocal images of FtsZ in the absence or presence of SBS showing the formation of FtsZ polymers. Samples were visualized after 30 min incubation (no GTP), and just after addition of GTP (+GTP).

b) Images of FtsZ ± SBS in the presence of FZ95 or FZ100, as specified. All elements in the sample were incubated 30 min and visualized before (no GTP), immediately after GTP addition (+GTP) and also 30 min after the addition of this nucleotide.

c) Images of FtsZ without and with FZ95 or FZ100, incubated together for 30 minutes prior visualization, before and after GTP addition. Afterwards, SBS was added and the sample was visualized again.

Concentrations were 10 µM FtsZ and 1 µM SBS and labeled components (FtsZ-Alexa 488 and SBS-Alexa 647). Samples were prepared in crowding conditions with 150 g/L dextran and the final DMSO concentration was 1%. In all cases, polymerization was triggered by the addition of 0.5 mM GTP. Images are representative of 2 independent experiments.
